# GPCRs identified on mitochondrial membranes: New therapeutic targets for diseases

**DOI:** 10.1016/j.jpha.2024.101178

**Published:** 2025-01-02

**Authors:** Yanxin Pan, Ning Ji, Lu Jiang, Yu Zhou, Xiaodong Feng, Jing Li, Xin Zeng, Jiongke Wang, Ying-Qiang Shen, Qianming Chen

**Affiliations:** State Key Laboratory of Oral Diseases, National Clinical Research Center for Oral Diseases, Chinese Academy of Medical Sciences Research Unit of Oral Carcinogenesis and Management, West China Hospital of Stomatology, Sichuan University, Chengdu, 610041, China

**Keywords:** GPCRs, Mitochondria, RAS, 5-HT, Melatonin, Purinergic

## Abstract

G protein-coupled receptors (GPCRs) are the largest family of membrane proteins in eukaryotes, with nearly 800 genes coding for these proteins. They are involved in many physiological processes, such as light perception, taste and smell, neurotransmitter, metabolism, endocrine and exocrine, cell growth and migration. Importantly, GPCRs and their ligands are the targets of approximately one third of all marketed drugs. GPCRs are traditionally known for their role in transmitting signals from the extracellular environment to the cell's interior via the plasma membrane. However, emerging evidence suggests that GPCRs are also localized on mitochondria, where they play critical roles in modulating mitochondrial functions. These mitochondrial GPCRs (mGPCRs) can influence processes such as mitochondrial respiration, apoptosis, and reactive oxygen species (ROS) production. By interacting with mitochondrial signaling pathways, mGPCRs contribute to the regulation of energy metabolism and cell survival. Their presence on mitochondria adds a new layer of complexity to the understanding of cellular signaling, highlighting the organelle's role as not just an energy powerhouse but also a crucial hub for signal transduction. This expanding understanding of mGPCR function on mitochondria opens new avenues for research, particularly in the context of diseases where mitochondrial dysfunction plays a key role. Abnormalities in the phase conductance pathway of GPCRs located on mitochondria are closely associated with the development of systemic diseases such as cardiovascular disease, diabetes, obesity and Alzheimer's disease. In this review, we examined the various types of GPCRs identified on mitochondrial membranes and analyzed the complex relationships between mGPCRs and the pathogenesis of various diseases. We aim to provide a clearer understanding of the emerging significance of mGPCRs in health and disease, and to underscore their potential as therapeutic targets in the treatment of these conditions.

## Highlights

• GPCRs existing on mitochondrial membrane affect the physiological function of mitochondria.
• Dysfunction in mGPCRs is linked to cardiovascular disorders and neurodegenerative disorders.
• Targeting mGPCRs offers therapeutic potential for treating these conditions.

## Graphical abstract

**Figure:**
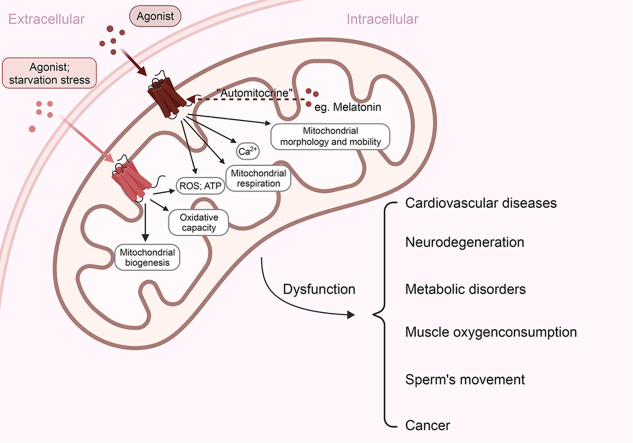

## 1 Introduction

G protein-coupled receptors (GPCRs) are a vast and versatile family of membrane proteins that play crucial roles in cellular communication and signal transduction. Traditionally, GPCRs have been associated with the plasma membrane by activating heterotrimeric G proteins, which are composed of three subunits: α, β, and γ. When an agonist (such as a hormone, neurotransmitter, or other signaling molecule) binds to a GPCR, the receptor undergoes a conformational change. This change enables the GPCR to act as a guanine nucleotide exchange factor (GEF) for the associated G protein. Specifically, the GPCR facilitates the exchange of guanosine diphosphate (GDP) for guanosine-5′-triphosphate (GTP) on the Gα subunit, activating it. The Gα subunit then dissociates from the Gβγ dimer, and both the Gα and Gβγ subunits can independently interact with various downstream effectors, such as enzymes or ion channels, to propagate the signal within the cell. This activation initiates a cascade of cellular responses, ranging from changes in gene expression to alterations in cell metabolism, ultimately influencing a wide array of physiological processes [1]. However, in recent years, a growing body of evidence has revealed that GPCRs are also present in intracellular compartments, including mitochondria. This discovery has expanded our understanding of GPCRs function, highlighting their involvement in regulating mitochondrial physiology and associated signaling pathways.

Mitochondria, as the “processors” of cells, are important organelles that produce energy through oxidative phosphorylation, but they are also key hubs for various cellular processes such as apoptosis, calcium homeostasis, and the production of reactive oxygen species (ROS). The localization of GPCRs on mitochondrial membranes suggests that they may directly affect these mitochondrial functions. In fact, mitochondrial GPCRs (mGPCRs) are not only involved in sensing a variety of intracellular and extracellular signals [2], but also have a profound impact on physiological and pathological changes in cells by regulating core mitochondrial functions such as ion uptake, oxidative phosphorylation, nitric oxide synthesis, apoptotic signaling, and ROS production. At present, the mechanism by which GPCRs locate to mitochondria is not fully understood. The study speculates that specific signal sequences or post-translational modifications may guide GPCRs to reach mitochondrial membranes [3]. Further research is needed to uncover these mechanisms and determine whether they are receptor-specific or general features of mitochondrial localization.

**Fig. 1:**
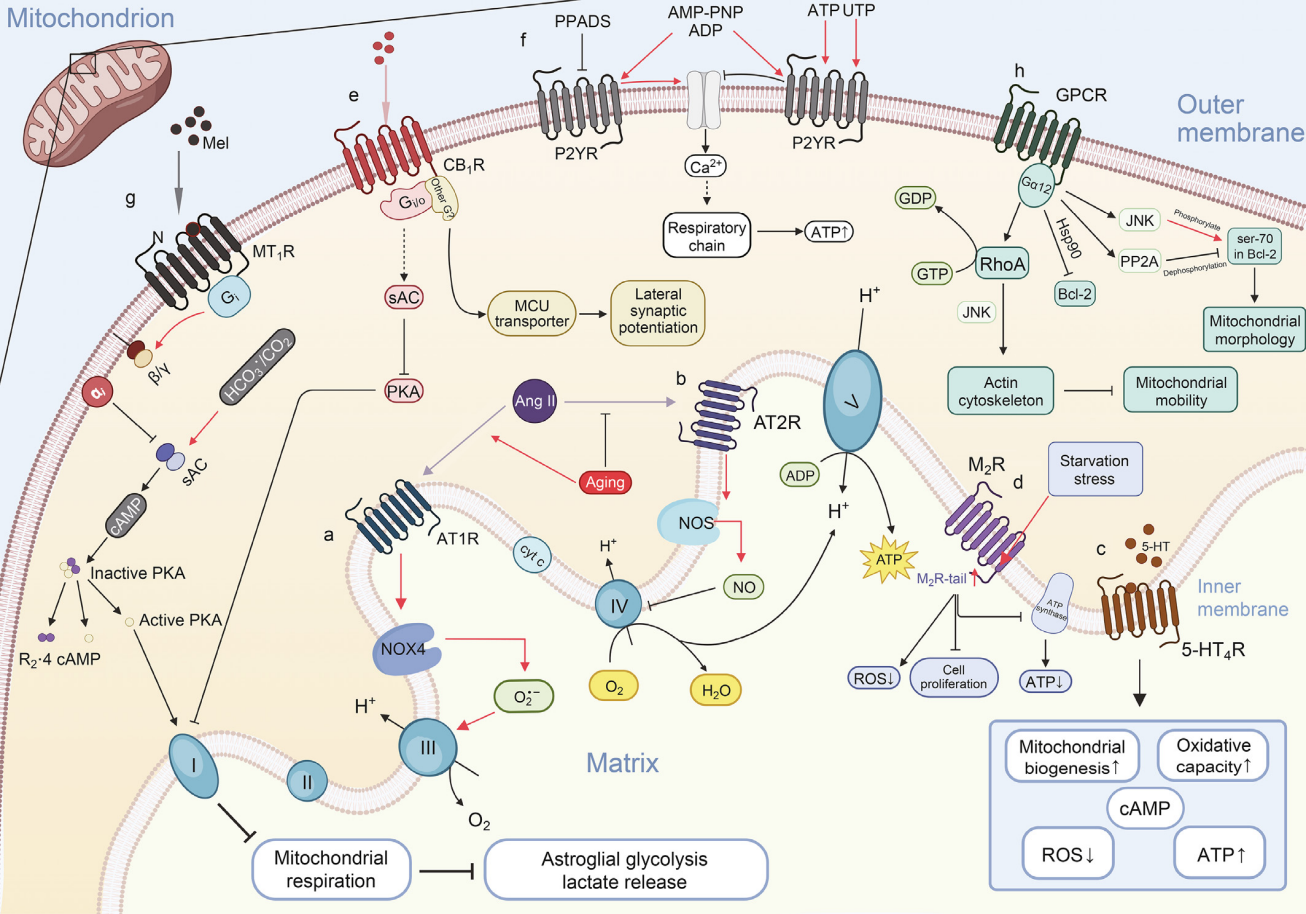
Seven G protein-coupled receptors (GPCRs) on mitochondria have an effect on the function of mitochondrion through various pathways. AT1R: angiotensin II type 1 receptor; AT2R: angiotensin II type 2 receptor; M_2_R: M_2_ muscarinic receptor; 5-HT_4_R: 5-hydroxytryptamine receptor 4; CB_1_R: cannabinoid type 1 receptor; P2YR: G protein-coupled P2Y receptor; Gα12: guanine nucleotide-binding protein subunit α-12; NOX4: NADPH oxidase 4; cyt c: cytochrome c; NOS: nitric oxide synthase; α_i_: inhibitory subunit of G_i_ protein; βγ: dissociated dimer of G protein; cAMP: cyclic adenosine 3′,5′-monophosphate; G_i_: inhibitory G protein; Mel: melatonin; PKA: protein kinase A; R_2_: dimer of regulatory PKA subunits; sAC: soluble adenylyl cyclase; MCU: mitochondrial calcium uniporter; PPADS: P2Y inhibitor(non-selective); RhoA: small guanosine triphosphatase Rho A; JNK: c-JunN-terminal kinase; PP2A: protein phosphatase 2A; Bcl-2: B-cell lymphoma-2; ROS: reactive oxygen species; GDP: guanosine diphosphate; GTP: guanosine-5'-triphosphate; AMP: adenosine monophosphate; ATP: adenosine triphosphate; UTP: uridine triphosphate; AMP-PNP: adenylyl imidodiphosphate; ADP: adenosine diphosphate; MT_1_R: melatonin receptor type 1. Created with BioRender.

At present, a number of specific mGPCRs have been identified and characterized, revealing their unique roles in mitochondria. For example, the β2-adrenergic receptor (β2AR) has been localized to the mitochondrial membrane and is involved in mitochondrial respiratory regulation and prevention of apoptosis. Similarly, the angiotensin II type 1 receptor (AT1R) is found in mitochondria and is thought to contribute to mitochondrial oxidative stress and dysfunction, particularly in the context of cardiovascular disease [4]. 5-hydroxytryptamine (5-HT) receptor [5], M_2_ muscarinic receptor (M_2_R) [3], cannabinoid receptor [6,7], melatonin receptors [8] and purinergic receptors [9] are all expressed in the mitochondria of nerve cells, which regulate the energy metabolism of nerve cells and neurons by participating in the regulation of intracellular metabolic activities, mitochondrial Ca^2+^ homeostasis, ROS production and other physiological processes, and play an important mechanism role in some neuron-related diseases (Fig. 1).

Continuing research to discover and validate other mGPCRs, and to determine the ligand-specific and functional significance of mGPCRs as a component of mitochondrial sensing machines will help us better understand their role in disease and may reveal new therapeutic strategies.

## 2 Seven GPCRs on mitochondria

### 2.1 Inner mitochondrial membrane (IMM)

#### 2.1.1 Angiotensin receptors

The renin-angiotensin system (RAS) is an important hormonal system in the human body that is closely related to various physiological and pathological states. It consists of renin, angiotensin-converting enzyme (ACE), angiotensinogen, and angiotensin II (Ang II). Ang II is the primary effector peptide of RAS, acting mainly through two types of angiotensin receptors: angiotensin II type 1 receptors (AT1R) and angiotensin II type 2 receptors (AT2R). These receptors have been identified in the nucleus and mitochondria and are both GPCR ligands responsible for transducing Ang II signals that cause blood vessel constriction. They are present in various tissues, including vascular smooth muscle, endothelium, heart, brain, kidneys, adrenal glands, and adipose tissue.

When activated by angiotensin II, AT1R promotes physiological functions through various pathways [10]. However, abnormal activation of AT1R leads to numerous pathophysiological conditions, including cardiovascular remodeling and hypertrophy, vascular inflammation and atherosclerosis, endothelial dysfunction, oxidative stress, extracellular matrix deposition, insulin resistance, angiogenesis, cancer, auto-antibody production, and malignant hypertension. Under normal conditions, AT2R expression is kept low, but it is significantly upregulated in disease states such as renal failure, vascular injury, and myocardial infarction to provide endogenous protection [11]. Research by Abadir et al. [12] has shown that functional AT2R exists in the inner mitochondrial membrane and co-locates with endogenous Ang II and Ang III. They also demonstrated that the activation of the mitochondrial angiotensin system is associated with mitochondrial nitric oxide production, which regulates respiration. Dysfunction of AT1R and AT2R in mitochondria is linked to cardiovascular diseases such as hypertension, stroke, and heart failure. AT2R interacts with AT1R in the cytoplasm, playing multiple roles in vascular remodeling. In most cases, Ang II binding to AT2R produces effects opposite to AT1R signaling, leading to its consideration as a counter-regulatory receptor within RAS [13]. AT2R expression is frequently upregulated in pathological states related to cardiovascular disease [[14], [15], [16]]. When AT1R is blocked, AT2R activation helps prevent aortic problems, making it a potential therapeutic target for vascular diseases caused by AT1R antagonists [17]. Wilson et al. [18] demonstrated that using ACE inhibitors or AT1R blockers can effectively reduce kidney damage in conditions like hypertension, diabetes, and heart failure. At the same time, AT2R stimulation also has shown significant effects on tissue repair and cell differentiation, and promotes diuresis and natriuria [19]. Additionally, it benefits the brain, lungs, heart, blood vessels, kidneys, pancreas, and skin [20].

With increasing age, mitochondrial AT1R levels rise while mitochondrial AT2R levels decrease, indicating an imbalance between mitochondrial angiotensin receptors [4]. These results are consistent with previous studies [21], which showed that AT2R is highly expressed in fetuses and newborns, but its content gradually decreases with age. However, research by Yu et al. [22] suggests that AT2R expression is higher in the brainstem, liver, and kidney tissues of adult rats compared to fetuses or neonates, possibly due to physiological differences between humans and rats.

In summary, the presence of a functional angiotensin system in mitochondria provides a foundation for understanding interactions between mitochondria and chronic disease states, revealing potential therapeutic targets to optimize mitochondrial function and reduce the burden of chronic diseases.

#### 2.1.2 5-HT receptor

5-Hydroxytryptamine, also named serotonin, is commonly found in the synaptic gap as a neurotransmitter and exerts its physiological effects primarily through 5-HT receptors, which influence a wide variety of physiological processes and cognitive functions in the brain. 5-HT receptors are a group of GPCRs and ligand-gated ion channels, which have been categorized into seven subfamilies (5-HT_1_ to 5-HT_7_), each encoding for one or more of the genes. Except 5-hydroxytryptamine receptor 3 (5-HT_3_R), all other 5-HT receptors are GPCRs that activate the intracellular second messenger cyclic adenosine monophosphate (cAMP) to produce excitatory or inhibitory effects. These receptors have been targeted with different drugs for the treatment of neurological and psychiatric disorders such as depression, bipolar disorder, psychosis, and obsessive-compulsive behavior.

Previous studies have shown that the 5-HT transporter protein (5-HTT) is expressed in cardiac cells and that environmental 5-HT concentrations affect cardiac cells [23]. Wang et al. [5] demonstrated that 5-hydroxytryptamine receptor 4 (5-HT_4_R) is located in cardiac mitochondria and is implicated in cardiovascular pathogenesis. 5-HT_4_R is functionally expressed in mitochondria and is involved in mitochondrial function and regulation to maintain mitochondrial Ca^2+^ homeostasis, ROS, and adenosine triphosphate (ATP) production efficiencies in response to stress and O_2_ tension. Lack of 5-HT_4_R in cardiomyocytes significantly affects mitochondrial Ca^2+^ uptake function under varying oxygen conditions, leading to increased cellular damage in response to hypoxia and alterations in cardiac rhythm. Interestingly, the effects of 5-HT_3_R and 5-HT_4_R on mitochondrial respiration were opposite: 5-HT_3_R knockdown increased the respiratory control ratio (RCR), while 5-HT_4_R knockdown led to a decrease. Furthermore, activation of both 5-HT_3_R and 5-HT_4_R significantly inhibited the opening of the mitochondrial permeability transition pore (mPTP). Furthermore, Tempio et al. [24] demonstrated that the 5-hydroxytryptamine receptor 7 (5-HT_7_R) is also expressed in the mitochondrial membrane of SH-SY5Y cells by testing the effect of the 5-HT_7_R antagonist (inverse agonist) SB-269970 on cytochrome c oxidase activity.

Research on 5-HT receptors on mitochondria presents exciting prospects for understanding cellular and cardiac physiology. The nuanced role of different 5-HT receptors, such as 5-HT_3_R and 5-HT_4_R, in modulating mitochondrial functions like calcium uptake and respiration under various conditions offers a promising avenue for exploring new therapeutic strategies. Further studies could elucidate how these receptors contribute to mitochondrial dynamics and energy metabolism, particularly in stress conditions such as hypoxia.

#### 2.1.3 M_2_ muscarinic receptor

The M_2_R is one of the five subtypes of muscarinic acetylcholine receptors (M_1_–M_5_), which are GPCRs [25]. M_2_R is primarily associated with the parasympathetic nervous system and plays a crucial role in regulating various physiological functions. Recent studies have shown that M_2_ receptor not only regulates extracellular signal transduction in the plasma membrane, but also locates in the inner mitochondrial membrane through *C*-terminal fragment (M_2_tail) to regulate and affect the physiological activities and functions of mitochondria.

The M_2_tail is a fragment of the M_2_ muscarinic receptor, specifically comprising the carboxyl-terminal (*C*-terminal) portion of the receptor, with its own *C*-terminal domain facing the matrix. Unlike the full M_2_ receptor, which functions as a GPCR at the plasma membrane to mediate acetylcholine signaling, M_2_tail is produced via an internal ribosome entry site (IRES)-mediated cap-independent translation mechanism, and influences mitochondrial oxidative phosphorylation by reducing oxygen consumption and ROS formation under stress conditions. This non-canonical role of the M_2_R's *C*-terminus highlights its regulatory function in cell respiration, contributing to cellular responses during metabolic stress [3]. However, much more research is still needed to figure out if factors besides starvation stress can regulate the expression of the M_2_R-tail protein, and what are the specific regulatory mechanisms [26].

Overall, this finding hints at a new regulatory mechanism within mitochondria that links M_2_R to the control of mitochondrial respiration and stress responses. Maybe through its ability to fine-tune mitochondrial respiration and ROS production, it can become a potential target for protective strategies in ischemia-reperfusion injury, cancer, neurodegenerative diseases, and more.

### 2.2 Outer mitochondrial membrane (OMM)

#### 2.2.1 Cannabinoid receptor

Cannabinoid type 1 (CB_1_) and cannabinoid type 2 (CB_2_) are seven-transmembrane GPCRs [27] initially identified as the cellular targets of exogenous cannabinoids, such as those derived from plants like Δ9-tetrahydrocannabinol (THC), or synthetic cannabinoids. This discovery suggested the presence of endogenous ligands, later identified as lipid derivatives of arachidonic acid, known as endo-cannabinoids.

CB_1_ receptors are highly concentrated in neuronal plasma membranes, where they play a critical role in regulating neuronal activity, metabolism, and functions [28]. Early studies indicated that THC, a cannabinoid derived from Cannabis sativa (marijuana), could impact mitochondrial functions [29], this process involves the dose-dependent binding of lipophilic agonists, such as THC, to mitochondrial CB_1_ (mtCB_1_) receptors located on the outer mitochondrial membrane, triggering intracellular signaling pathways that regulate mitochondrial function [30]. Bénard et al. [31] demonstrated that CB_1_ receptors are located on neuronal mitochondrial membranes and that their activation directly regulates mitochondrial energetics [32,33]. Other study also finds that mtCB_1_ receptor was shown to interact with an intramitochondrial Gαi protein that inhibits the soluble adenylyl cyclase (sAC) in the matrix which is activated by CO_2_/HCO_3_^−^, and this process further inhibits activity of the citric acid cycle stimulates electron throughput and, thus, affects respiration in the electron transfer chain (ETC) [[34], [35], [36]]. Beyond neurons, recent evidence also points to the role of CB_1_ receptor signaling in regulating mitochondrial biogenesis in peripheral non-neural tissue. For instance, mtCB_1_-induced reductions in ROS result in hypoxia-inducible factor 1α (HIF1α) destabilization and impair glycolysis in astrocytes, leading to decreased atroglial glycolysis and lactate release, which may cause a decline in neuronal bioenergetics [37]. Additionally, functions, such as sperm movement [38,39], muscle oxygen consumption regulation [6,30], and progesterone production [40], are associated with mtCB_1_ receptor activity. The concentration of endocannabinoid CB_1_ receptor agonists also decreases atrial muscle contractility in a concentration-dependent manner [41].

Research into the intramitochondrial signaling of the mtCB_1_ receptor is still in its early stages and has already revealed the complexity of this pathway. However, the precise mechanism by which this GPCR targets the mitochondrial membrane and whether it can translocate to other subcellular locations remains an open question that requires further experimental investigation.

#### 2.2.2 Purinergic receptor

Purinergic signaling is a communication system in the body that involves purines, such as adenosine and ATP, as signaling molecules. This system is crucial for various physiological functions, including neurotransmission, inflammation, and the regulation of blood flow. Purinergic receptors are proteins found on the surface of cells that respond to purine nucleotides and nucleosides, converting extracellular nucleotide signals into cellular responses. These receptors, which include P1 receptors (adenosine receptors) and P2 receptors (further divided into P2X ionotropic receptors and P2Y G protein-coupled receptors), are expressed on a wide variety of cells. They detect changes in extracellular purine concentrations and transduce these signals into the cell, leading to various outcomes such as activation of intracellular signaling cascades, changes in ion permeability, and modulation of gene expression.

P2Y receptor, members of the GPCR family, have a close connection with mitochondria. Belous et al. [42] reported that the presence of P2Y_1_-like and P2Y_2_-like receptors in hepatocyte mitochondria (designated as mP2Y_1_ and mP2Y_2_). These mitochondrial receptors sense and respond to cytoplasmic purine levels (ATP, adenosine diphosphate (ADP), and adenosine monophosphate (AMP)) via the purinergic GPCRs P2Y_1_ and P2Y_2_. In hepatocytes, experiments proof that activation of mitochondrial P2Y_1_ stimulates Ca^2+^ uptake, while activation of P2Y_2_ inhibits it. Although the precise sub-organellar locations of these receptors are still unclear, they are believed to be coupled to phospholipase C (PLC) and involved in downstream regulation of the mitochondrial calcium uniporter (MCU). Other researches also support this view [9,43]. Additionally, P2Y_2_ receptor activation may be involved in cell migration and invasion processes by influencing mitochondrial dynamics and morphology.

P2Y_1_ and P2Y_2_ receptors also play significant roles in the nervous system. Studies show that these receptors are present in rat astrocytes [7], where they influence neural activity and nerve fiber growth while controlling microglia migration [44]. Overactivation of the P2Y_1_ receptor and dysfunction of the P2Y_2_ receptor may contribute to neuroinflammation and neurodegeneration, such as in the pathological course of Alzheimer's disease (AD), where enhanced P2Y_1_ receptor expression is associated with astrocyte activation, potentially leading to cognitive decline [45].

In general, P2Y_1_ and P2Y_2_ receptors are closely related to mitochondrial function through different signal transduction pathways, and participate in the regulation of intracellular metabolic activities, Ca^2+^ ion balance, ROS production and other important physiological processes. In pathological states such as neurodegenerative diseases, the interaction between P2Y receptor and mitochondrial function may play an important role in the occurrence and development of diseases.

#### 2.2.3 Melatonin receptor

Melatonin, a hormone primarily secreted by the pineal gland, plays a crucial role in regulating circadian rhythms and exhibits potent neuroprotective properties. Its physiological effects are largely mediated through GPCRs, specifically the melatonin receptor subtypes MT_1_ and MT_2_.

These receptors are widely distributed across various tissues, including the brain, and are involved in modulating numerous biological processes such as sleep, mood, and immune function. When melatonin binds to these GPCRs, it activates intracellular signaling pathways that include the inhibition of adenylate cyclase, reduction of cyclic AMP levels, and modulation of calcium signaling. These actions lead to diverse cellular responses, such as antioxidant effects, inhibition of apoptosis, and regulation of mitochondrial function. The interaction between melatonin and its GPCRs underscores its therapeutic potential in treating sleep disorders, neurodegenerative diseases, and other conditions associated with oxidative stress and mitochondrial dysfunction.

Melatonin exhibits strong neuroprotective properties [[46], [47], [48], [49]], such as inhibiting mitochondrial cytochrome c release, preventing caspase activation [[50], [51], [52]], and reducing ROS levels after ischemia events [53]. Although the precise mechanisms behind melatonin's neuroprotection are not fully understood, research by Suofu et al. [54] indicates that lipophilic melatonin is produced in the mitochondrial matrix exclusively and release into the cytosol where it binds to high-affinity MT_1_ receptors on the outer mitochondrial membrane through organelle-based intracellular receptor–ligand (i.e., automitocrine) pathway and activates the MT_1_/G protein signaling system. This activation inhibits cytochrome c release, thereby blocking caspase activation and preventing neurodegeneration. Melatonin's antioxidant and radical scavenging activities further contribute to reducing oxidative damage [55]. In mouse brain neurons, mitochondrial MT_1_ receptors located on the outer membrane, in conjunction with adenylate cyclase in the membrane space, inhibit permeability transition and cytochrome c release, thereby protecting against ischemic injury and cell death [53]. Studies also indicate that melatonin inhibits cytochrome c release in neurons exposed to H_2_O_2_, and that the MT_1_ receptor antagonist luzindole blocks this protective effect [8,51].

Cells are highly permeable to melatonin, which can activate both plasma membrane and mitochondrial receptors [56]. Researchers have developed a light-activated melatonin ligand targeting mitochondria, demonstrating that melatonin inhibits mitochondrial respiration via the MT_1_ receptor. This neuroprotective effect has been observed in mouse models of newborn hypoxic–ischemic (H–I) brain injury, where it involves the restoration of MT_1_ receptors, inhibition of mitochondrial cell death pathways, and suppression of astrocytic and microglial activation [57]. Additionally, melatonin-induced cardio protection may depend on receptor activity, with anti-adrenergic actions mediated by nitric oxide synthase (NOS) and guanylyl cyclase activation playing significant roles. Additional studies are needed to clarify the effects of MT_1_ signaling on mitochondria and the plasma membrane in various cell types.

#### 2.2.4 Others

GPCRs located on mitochondrial membranes can be internalized upon activation, potentially enabling signaling in spatially distinct manners [58]. For instance, the β subunit of heterotrimeric G proteins, specifically guanine nucleotide-binding protein β subunit 2 (Gβ2), a WD40 repeat protein, plays a crucial role in mitochondrial fusion. Gβ2 is enriched on the mitochondrial surface and interacts specifically with mitofusin 1 (Mfn1) to regulate mitochondrial fission and fusion [59]. Additionally, guanine nucleotide-binding protein subunit α-12 (Gα12), one of the four families of α subunits in heterotrimeric G proteins, can specifically target mitochondria and is involved in controlling mitochondrial morphology and dynamics [60].

Moreover, G-protein coupled receptor 35 (GPR35), including the canine urinary quinolinic acid-activated variant, may translocate to the OMM under stress conditions, where it regulates the oxidative phosphorylation (OxPhos) system and plays a role in ischemic protection by modulating mitochondrial function [61].

## 3 Discussion and perspective

GPCRs are a vast and diverse family of membrane proteins that play a crucial role in transmitting signals from the extracellular environment to the inside of the cell [62]. Traditionally, GPCRs have been studied extensively in the context of their presence on the plasma membrane, where they mediate various physiological processes, including neurotransmission, hormone release, and immune responses. However, emerging evidence has revealed that GPCRs are also present on the membranes of intracellular organelles [63], particularly mitochondria, where they serve distinct and critical functions.

Mitochondria, which are double-membrane-bound organelles, serve as a key location for GPCRs localization. The OMM faces the cytoplasm, while the inner membrane IMM forms folds that protrude into the mitochondrial matrix. mGPCRs are typically found on the OMM, but under certain conditions, they may also localize to the IMM. Although the precise mechanisms determining the localization of GPCRs on these mitochondrial membranes remain unclear, it is generally believed that the *N*-terminal extracellular domain of GPCRs on the OMM is oriented towards the cytoplasm, allowing interactions with cytoplasmic G proteins and other effectors. The *C*-terminal intracellular domain, meanwhile, interacts with the intermembrane space (IMS). This structural arrangement enables these GPCRs to receive and transmit signals from the cytoplasmic environment. And another way to locate maybe is to use pathways similar to those of other outer membrane proteins, like the translocase of the outer membrane (TOM) complex [64,65]. In contrast, IMM-localized GPCRs have ligand-binding domains facing either the IMS or the matrix, where they interact with IMM-resident signaling proteins. Such interactions play critical roles in regulating mitochondrial functions, including oxidative phosphorylation, calcium homeostasis, and ROS production. The presence of GPCRs in mitochondria varies by tissue, depending on the specific type of GPCR and its associated signaling pathways. For example, GPR35 [61], which is activated by quinolinic acid, is found in mitochondria of certain tissues like the brain and is known to translocate to the OMM under stress conditions.

Both extracellular and intracellular signals or agonists can interact with mGPCRs in ways that are similar to, yet distinct from, their interactions at the plasma membrane. This interaction can be influenced by three key factors: receptor interactions, lipid composition, and signaling pathways. First, mGPCRs, like those at the plasma membrane, initiate, regulate, or terminate signaling pathways by binding to specific molecules and proteins within the cell, with this binding often being highly specific and concentration-dependent [30]. Second, the lipid environment of the mitochondrial membrane differs from that of the plasma membrane, potentially altering receptor conformation [66], ligand affinity, and membrane permeability. Besides extracellular stimuli, some agonists may be secreted within mitochondria and act through an autocrine-like pathway [54]. Finally, the distinct downstream effectors present in the cytoplasm and mitochondria may lead to different signal transduction outcomes, even if the same receptor-ligand interaction occurs. This variability in outcomes may, in turn, influence the receptor's ligand-binding capacity upstream. Further understanding of these mechanisms and their tissue-specific roles is crucial for exploring receptor localization's physiological effects and for identifying potential therapeutic targets in various diseases.

mGPCRs play crucial roles in various cellular processes, distinct from their functions at the plasma membrane. One key role of GPCRs within mitochondria is regulating mitochondrial dynamics, including fusion, fission, and biogenesis. For instance, while β_2_ adrenergic receptor (β_2_AR) at the plasma membrane traditionally regulates cardiovascular functions, smooth muscle relaxation, and lipolysis through stimulatory G (Gs) protein activation and cAMP production [67], its mitochondrial localization allows it to influence mitochondrial dynamics and energy production [68]. Specifically, mitochondrial β_2_AR modulates fusion and fission, thereby affecting overall mitochondrial health and function. Another important function of mGPCRs is in regulating cellular energy metabolism. Mitochondrial AT1R influences ROS generation and can activate mitochondrial apoptotic pathways, indicating its role in oxidative stress and apoptosis independently of its plasma membrane actions. In contrast, when AT1R localizes to the cell surface, it mediates vasoconstriction, blood pressure regulation, and inflammation. Its activation triggers downstream signaling cascades involving protein kinase C (PKC) and ROS production. Mitochondrial 5-HT receptor also contributes to energy metabolism, ROS regulation, and calcium handling within mitochondria, with implications for metabolic regulation and neuroprotection—distinct from its plasma membrane role. Additionally, mGPCRs are involved in regulating apoptosis. For example, GPCRs that facilitate calcium ion entry into mitochondria can influence the release of pro-apoptotic factors like cytochrome c, thereby controlling the intrinsic pathway of apoptosis.

Emerging research indicates that GPCRs located on the plasma membrane can also influence mitochondrial functions through intracellular communication pathways, significantly affecting metabolism, oxidative stress, and calcium homeostasis. GPCRs, such as those associated with the G protein subunit alpha 13 (Gα13) subunit, can modulate mitochondrial activity by controlling oxidative stress and regulating key mitochondrial enzymes and antioxidant pathways. For instance, activation of Gα13-linked GPCRs has been linked to enhanced expression of mitochondrial superoxide dismutase (SOD2), a key enzyme in reducing oxidative damage. This regulation helps maintain cellular balance under stress conditions and promotes adaptive metabolic responses [69]. Calcium signaling is another crucial pathway where plasma membrane-mitochondrial communication plays a role. GPCRs can modulate calcium ion transport into mitochondria via interactions with specific membrane-associated contact sites, such as mitochondria-associated membranes (MAMs). This calcium exchange is vital for maintaining mitochondrial energy production and regulating apoptosis [70].

**Fig. 2:**
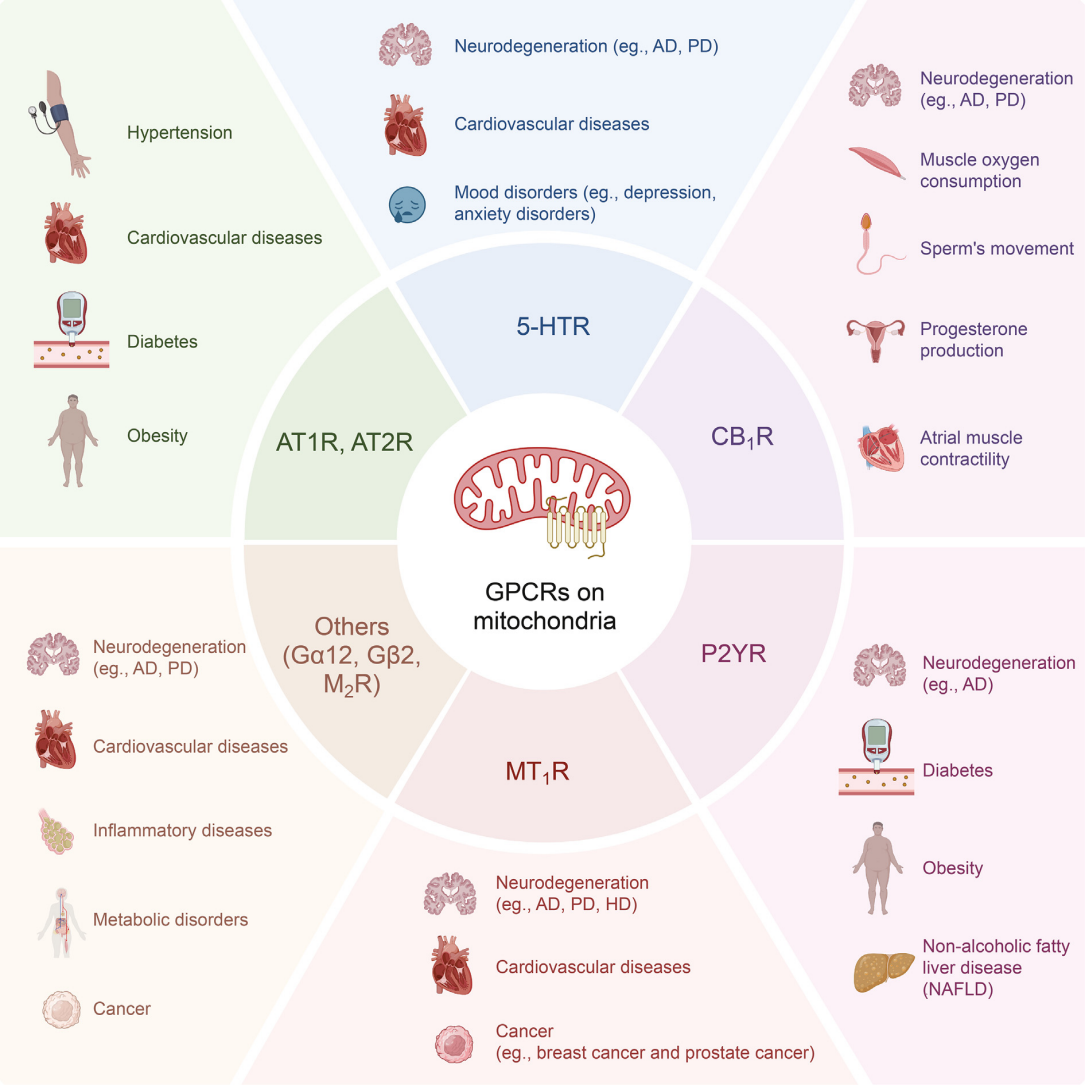
Diseases associated with abnormal mitochondrial G protein-coupled receptors (GPCRs). A variety of diseases associated with dysregulation of GPCR signaling on mitochondria. AT1R: angiotensin II type 1 receptor; AT2R: angiotensin II type 2 receptor; M_2_R: M_2_ muscarinic receptor; 5-HT_4_R: 5-hydroxytryptamine receptor 4; CB_1_R: cannabinoid type 1 receptor; P2YR: G protein-coupled P2Y receptors; Gα12: guanine nucleotide-binding protein subunit α-12; Gβ2: guanine nucleotide-binding protein β subunit 2; GPR35: G-protein coupled receptor 35; AD: Alzheimer's disease; PD: Parkinson's disease; HD: Huntington's disease. Created with BioRender.

Dysregulation of mGPCRs is increasingly recognized as a contributing factor in the pathogenesis of various diseases. In neurodegenerative disorders such as AD [44]and Parkinson's disease (PD) [71,72], abnormal GPCRs signaling within mitochondria disrupts calcium homeostasis and mitochondrial function, leading to neuronal death and disease progression. For example, the altered function of GPR35 in mitochondria has been linked to impaired energy metabolism and increased oxidative stress in neurons, exacerbating neurodegeneration [61]. Similarly, in cardiovascular diseases [73], dysregulated GPCRs signaling in mitochondria can lead to mitochondrial dysfunction, contributing to conditions such as heart failure. Moreover, certain types of cancer exhibit altered mGPCRs signaling, which may support cancer cell proliferation and survival by enhancing mitochondrial biogenesis and resistance to apoptosis. In metabolic disorders, including diabetes, aberrant GPCRs activity in mitochondria can lead to insulin resistance and impaired energy metabolism, further complicating disease management (Fig. 2).

Given their crucial roles in mitochondrial function and their involvement in disease, m GPCRs represent promising therapeutic targets. Developing drugs that specifically modulate GPCRs activity within mitochondria could offer novel treatment strategies for a range of conditions [63]. For instance, selective agonists or antagonists that target mGPCRs could potentially restore normal mitochondrial function in neurodegenerative diseases, thereby slowing disease progression and improving patient outcomes. In cardiovascular diseases, therapies aimed at correcting m GPCRs signaling could help preserve mitochondrial integrity and function, reducing the risk of heart failure. Furthermore, targeting mGPCRs in cancer cells could enhance the efficacy of existing treatments by promoting apoptosis and inhibiting tumor growth. However, how to accurately identify and locate mGPCRs is another challenge. Researchers have developed mitochondria-targeted ligands by conjugating them with lipophilic cations, such as triphenylphosphonium (TPP+) [74]. This strategy enhances the accumulation of these compounds in the mitochondria due to the organelle's membrane potential. Selective ligands with specific modifications can help distinguish between the effects of plasma membrane and mGPCRs. Additionally, creating mutant GPCRs or expressing tagged receptors with mitochondrial localization sequences (such as mitochondrial targeting sequences, MTS) allows for a comparison of signaling outcomes [75]. Using RNA interference or CRISPR/Cas9 systems to target GPCRs specifically within mitochondria can also help isolate the role of these receptors. And a common experimental approach involves using ligands to selectively block or activate receptors at the cell surface, ensuring that any observed mitochondrial effects are indeed due to activation of intracellularly localized GPCRs. As a more realistic example, Gβ subunits have traditionally been considered unstable and nonfunctional if not paired with a Gγ subunit. However, there is emerging evidence that Gβ2 may exhibit stability and functionality independent of Gγ in specific cellular contexts, such as mitochondria [59]. This suggests that monomeric Gβ2 may have unique, more selective binding partners within mitochondria, perhaps interacting with proteins that are unique to mitochondrial signaling. This difference may therefore suggest a specific targeting or import mechanism that bypasses these membrane lipid interactions, which could provide key insights into non-canonical G protein signaling pathways and reveal new therapeutic targets, particularly for diseases where mitochondrial dysfunction plays a key role. In the future, further research is needed to fully elucidate the specific mechanisms by which GPCRs operate within mitochondria and their broader implications in health and disease.

## 4 Conclusion

In conclusion, mGPCRs play essential roles in cellular function and are involved in the pathogenesis of various diseases. Their tissue-specific distribution, involvement in critical mitochondrial processes, and association with disease make them attractive targets for future therapeutic interventions. Our improved understanding of mitochondrial GPCR signaling has provided tremendous impetus for the continued exploration of targeted therapeutic approaches for diseases associated with mitochondrial dysfunction.

## CRediT authorship contribution statement

**Yanxin Pan:** Writing – original draft, Data curation. **Ning Ji:** Methodology. **Lu Jiang:** Methodology. **Yu Zhou:** Methodology. **Xiaodong Feng:** Supervision, Methodology. **Jing Li:** Methodology. **Xin Zeng:** Methodology. **Jiongke Wang:** Methodology. **Ying-Qiang Shen:** Methodology, Conceptualization. **Qianming Chen:** Writing – review & editing, Supervision, Funding acquisition, Concept-ualization.

## Declaration of competing interest

The authors declare that there are no conflicts of interest.
